# Breast Cancer Survivors’ Attitudes toward eMental Health: A Cross-Sectional Study

**DOI:** 10.3390/healthcare11131920

**Published:** 2023-07-03

**Authors:** Cristina Mendes-Santos, Teresa Campos, Diana Ferreira, Elisabete Weiderpass, Rui Santana, Gerhard Andersson

**Affiliations:** 1 Fraunhofer Portugal AICOS, 4200-135 Porto, Portugal; 2 Department of Culture and Society (IKOS), Linköping University, 58183 Linköping, Sweden; 3 NOVA National School of Public Health, Public Health Research Centre, Universidade Nova de Lisboa, 1099-085 Lisbon, Portugal; ruisantana@ensp.unl.pt; 4 Faculty of Sports, University of Porto (FADEUP), 4099-002 Porto, Portugal; tcampos83@gmail.com; 5 Center for Psychology, University of Porto, 4200-135 Porto, Portugal; up202202196@up.pt; 6 International Agency for Research on Cancer (IARC), 69366 Lyon, France; director@iarc.who.int; 7 Department of Behavioural Sciences and Learning (IBL), Department of Biomedical and Clinical Sciences, Linköping University, 58183 Linköping, Sweden; gerhard.andersson@liu.se; 8 Department of Clinical Neuroscience, Psychiatry Section, Karolinska Institutet, 17177 Stockholm, Sweden

**Keywords:** attitudes, breast cancer, eMental Health, Portugal, EU

## Abstract

Background: Breast cancer survivors’ (BCS) attitudes toward eMental Health (eMH) are largely unknown, and adoption predictors and their interrelationships remain unclear. This study aimed to explore BCS’ attitudes toward eMH and investigate associated variables. Methods: A cross-sectional study involving 336 Portuguese BCS was conducted. Attitudes toward eMH, depression and anxiety symptoms, health-related quality of life, and sociodemographic, clinical, and internet-related variables were assessed using validated questionnaires. Spearman-ranked correlations, χ^2^, and multiple regression analyses were computed to explore associations between attitudes and collected variables. Results: BCS held a neutral stance toward eMH. In models adjusted for age and education, positive attitudes were statistically significantly associated with increased depressive symptoms and worse emotional, cognitive, and body image functioning. Social network use, online health information and mental healthcare seeking, higher self-reported knowledge of eMH, and previous use of remote healthcare were positively associated with better attitudes toward eMH. Conclusions: eMH programs targeting BCS seem to be a promising strategy for providing supportive psychosocial care to BCS. However, increasing awareness about eMH efficacy and security may be necessary to improve its acceptance and use among BCS. Additional research is necessary to understand how BCS’ unmet care needs, and specifically their psychological distress severity, may impact BCS’ acceptance and use of eMH.

## 1. Introduction

Breast cancer (BC) is the most diagnosed cancer worldwide. In 2020, 2.3 million incident cases were estimated and 7.8 million women had lived for at least 5 years after their breast cancer diagnosis [1]. In Portugal, approximately 7041 women were diagnosed in 2020, and 27,051 were alive within 5 years post-diagnosis [2].

Anxiety, depression, fear of cancer recurrence, fatigue, sleeping problems, and sexual dysfunction are common among BCS [3,4]. Such conditions are triggered by sequelae of treatment, late effects, and unmet care needs, which hinder BCS’ health-related quality of life (HRQoL) [5]. Despite the efficacy of psychosocial interventions in treating these conditions (e.g., cognitive-behavioral therapy (CBT)) [6], the treatment gap for mental health problems among BCS is wide. Underlying causes include the distance from healthcare services, lack of training of cancer specialists in mental health, constrained budgets, and a dominant model of in-person psychosocial care provision that hinders the diagnosis of mental disorders and the dissemination of treatment programs [7].

In this context, eMental Health (eMH)—the use of digital technologies to support mental healthcare delivery [8]—and particularly, internet interventions and self-help technology-enabled interventions [9]—are considered efficacious [10] and cost-efficient [11,12] strategies to provide mental healthcare to cancer survivors [13]. Previous reviews [13,14,15] suggest that internet interventions are feasible and efficacious in improving HRQoL, self-efficacy, depression, distress, and perceived stress in BCS [14]. Recently, various randomized controlled trials (RCT) targeting BCS attested to the efficacy of internet interventions in improving depression [16], anxiety [17], distress [18], fear of cancer recurrence [19], sleep quality [20,21], sexual functioning [22], and HRQoL [23,24] in this population. Despite its usefulness and cost-effectiveness, cancer-related internet intervention research is scarce [14], and implementation in cancer settings is limited [25].

Prior research has been performed to identify potential drivers of and barriers to internet interventions’ adoption and to characterize professionals’ and cancer survivors’ attitudes toward such interventions [25,26,27]. Most studies report that the attitudes of professionals and cancer survivors toward eMH range from neutral to positive [26,27,28]. However, few studies targeted BCS [29,30].

In these studies, eMH adoption was positively influenced by individuals’ motivational readiness to use and engage with internet interventions [31,32], unmet care needs [33,34,35], and the perception of internet interventions as a means of having increased access to supportive care or an alternative to traditional care. This has been observed predominantly in survivors presenting with sensitive symptoms [34,36] and valuing anonymity [37]. Interventions’ usefulness [34], ease of use [30,31,32,38], and self-paced delivery [31,32,38] were also documented as empowering [37] and expediting adoption in cancer survivors. Likewise, the timely, tailored [26], and flexible [37] delivery of interventions has been identified as an important adoption driver in this population. In contrast, cancer recurrence and the reporting of physical symptoms [31,32,36] deterred adoption by cancer survivors. Similarly, perceptions of internet interventions’ limited usefulness and difficulties in integrating such interventions into daily routines [32,34,35], lack of internet skills [39], and usability issues have been documented as barriers hindering implementation in cancer settings [26]. Mixed results have been published concerning the use of in-person support [26].

Despite these findings, cancer-related internet interventions’ adoption predictors and their interrelationships are still unclear [25,26,27], and BCS’ attitudes toward such interventions are largely unknown [26,29,37].

The aim of this study was to explore BCS’ attitudes toward eMH and investigate variables associated with these attitudes. The main research questions guiding this research were “What is the stance of BCS toward eMH?” and “What factors are associated with BCS’ attitudes toward eMH?”. The findings of this study will contribute to a better understanding of eMH attitudinal predictors in BCS and inform the development and implementation of eMH in cancer settings.

## 2. Materials and Methods

### 2.1. Study Design

A cross-sectional study design was used. The study was approved by the ethical committees of IPO-Porto; Centro Hospitalar Universitário do Porto; Centro Hospitalar S. João; ULS-Matosinhos; Hospital CUF Porto; and the Portuguese Data Protection Committee (approval 10727/2017). Written informed consent was obtained from all participants. This study adhered to the STROBE guidelines for reporting cross-sectional studies [40].

### 2.2. Procedures

A convenient sample of BCS was recruited from the Day Hospitals or Breast Clinics of five hospitals in Porto (north of Portugal) in 2019. Hospitals were selected according to their treatment standards and catchment areas. Women aged above 18 years, with a confirmed diagnosis of BC, and capable of reading/writing in Portuguese were eligible and invited to participate by local clinical teams or the researchers. Participant BCS were invited to fill in a paper-and-pencil or online questionnaire at iTerapi [41].

### 2.3. Measures

#### 2.3.1. Sociodemographic, Clinical, and Internet-Related Variables

Sociodemographic variables were collected using a self-developed questionnaire assessing: age; education; marital status; occupation; professional status; residence; distance between residence and cancer center; access and frequency of internet use; online health information and mental healthcare seeking; social networks use; eMH self-reported knowledge (i.e., a single item stating “I am familiar with the concept of psychological interventions carried out over the internet?” and scored using a 5-point Likert scale (0 = “Completely disagree” to 5 = “Completely agree”); and remote healthcare use (i.e., a single item assessing if participants ever received medical, nursing, or psychological support via the internet or the telephone and scored dichotomously (0 = “No”; 1 = “Yes”). The following clinical data were retrieved from participants’ medical records at each site using a standardized data abstraction form: time since diagnosis; type of BC; type of treatment performed; tumor, node, and metastasis status (TNM status); Eastern Cooperative Oncology Group (ECOG) performance status; and psychiatric history.

#### 2.3.2. Anxiety and Depression

Anxiety and depression symptoms were measured using the Generalized Anxiety Disorder (GAD-7) questionnaire [42] and the Patient Health Questionnaire (PHQ-9) [43], respectively. PHQ-9 is scored using a 4-point Likert scale, ranging from 0 (“Not at all”) to 3 (“Nearly every day”). The total score ranges from 0 to 27, with high scores correlating with greater severity of symptoms of depression. Cut-off points 5, 10, 15, and 20 represent the thresholds for mild, moderate, moderately severe, and severe depressive symptoms, respectively [43]. GAD-7 is scored using a 4-point Likert scale, ranging from 0 (“Not at all”) to 3 (“Nearly every day”). The total score ranges from 0 to 21, with high scores indicating greater severity of anxiety symptoms. The cut-off points 5, 10, and 15 categorize anxiety symptoms as normal (0–4), mild (5–9), moderate (10–14), and severe (15–21), respectively [42]. Both scales have been previously validated in Portuguese oncology settings (PHQ-9 α = 0.89; GAD-7 α = 0.88) [42,43].

#### 2.3.3. HRQoL

HRQoL was measured using EORTC QLQ-C30 (v. 3.0) and QLQ-BR23 [44,45].

EORTC QLQ-C30 is a self-reporting, multidimensional HRQoL measure composed of 30 items and nine multi-item scales, namely, six functional scales (physical, role, cognitive, emotional, and social), three symptom scales (fatigue, pain, and nausea/vomiting), and a global health status/HRQoL scale. The questionnaire also includes five single-item symptom measures assessing dyspnea, loss of appetite, insomnia, constipation, and diarrhea and an extra single-item measure evaluating the perceived financial impact of the disease. All the items are scored using a 4-point Likert scale extending from 1 (“not at all”) to 4 (“very much”), except for the two items assessing the global health status/HRQoL scale, which adopt a modified 7-point linear analog scale. A linear transformation should be performed to obtain standardized scores ranging from 0 to 100, with higher scores representing higher response levels, i.e., “better” level of functioning or “worse” level of symptoms.

EORTC QLQ-BR23 comprises 23 items and five multi-item scales, namely, two functional scales (body image and sexual functioning) and three symptom scales (arm and breast symptoms and systematic therapy side effects). Additionally, single-item measures assess sexual enjoyment, future perspective, and being upset due to hair loss. The scoring method for the QLQ-BR23 is similar to that for the functional and symptom scales/single items of the QLQ-C30, i.e., all scores range from 0 to 100. A high score for the functional scales corresponds to a high/healthy level of functioning, whereas a high score for the symptom scales represents a high level of symptomatology or problems. QLQ-BR23 has been clinically and cross-culturally validated, evidencing high internal consistency. QLQ-C30 and QLQ-BR23 have been validated for the Portuguese population [44,45].

#### 2.3.4. Attitudes toward eMH

The Attitudes Toward Internet Interventions Survey (ATIIS) [46] measured BCS’ attitudes toward eMH. ATIIS is a self-developed questionnaire characterizing clients’ and therapists’ use of and attitudes toward eMH. The questionnaire has two versions tailored to psychologists and clients. Whereas the psychologists’ version has already been validated for the Portuguese population (α= 0.91) the clients’ version was adapted and validated to BCS in the context of this research (Table S1).

The ATIIS BCS version is a self-reporting, 34-item questionnaire that assesses: (1) the use of digital technology for healthcare purposes, (2) eMH self-reported knowledge, and (3) attitudes toward eMH. The attitudes section is composed of 16 items clustering in two dimensions, labeled as “positive” and “negative” attitudes (α = 0.93). Items are scored using a 5-point Likert scale (0 = “Completely disagree”; 5 = “Completely agree”). Higher summated scores represent a better attitude toward eMH. A detailed description of ATIIS’ attitudes section development and psychometric assessment is provided in File S1.

### 2.4. Analysis

All analyses were performed using IBM SPSS Statistics v.27.0. Hypothesis tests were conducted at a confidence level of 95% with a *p*-value of 0.05.

First, descriptive statistics, namely medians, interquartile ranges, counts/percentages, and percentiles were computed to describe the study sample, report on participants’ eMH use and eMH self-reported knowledge, and examine self-reported measures’ scores. Next, associations between attitudes and sociodemographic, clinical, and internet-related variables, as well as anxiety, depression, and HRQoL were investigated. Spearman rank correlations were used to explore associations between continuous variables and continuous attitudes scores. χ^2^ analyses and post hoc tests were used to assess if categorical variables would be associated and if there were differences between participant BCS holding extreme attitudes (i.e., scoring <Q1 or >Q3 in the ATIIS attitudes section). Finally, multiple regression analyses were performed to further examine the relationships between associations previously identified as statistically significant, while adjusting for age and education [47]. The enter method was used for this purpose, as we assumed all variables could have equal importance in the developed models. All preliminary assumptions of regression analysis were met.

## 3. Results

### 3.1. Participants’ Characteristics

**Figure 1:**
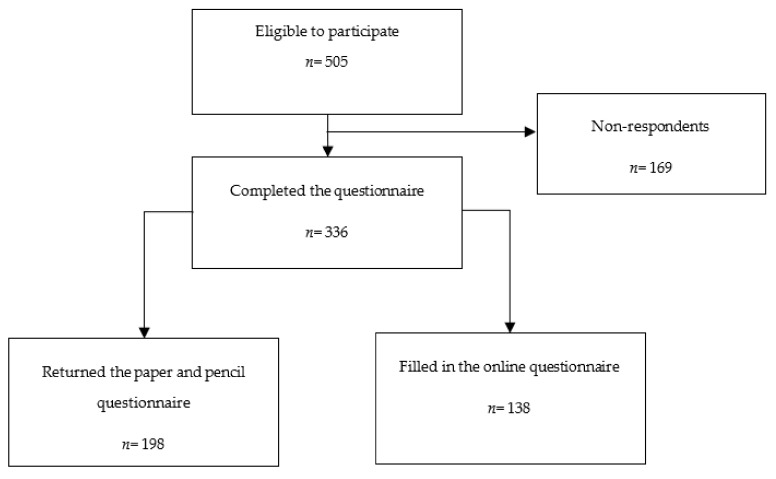
Recruitment flow diagram.

A total of 505 women were invited to participate in the study. Of these, 169 did not return the survey, and 336 responded to the survey (67% response rate) (Figure 1).

The median age of our sample was 53 years (IQR = 15; Min: 26; Max: 82). Most participants were married/in de facto relationships (67%; *n* = 225) and 32% (*n* = 107) were professionally active. The majority of participant survivors were diagnosed with an invasive ductal carcinoma NST (73%; *n* = 231), Luminal B HER2-negative (33%; *n* = 88), or Luminal B HER2-positive (31%; *n* = 82), two years before the study (Range: 0–24 months). Forty-nine percent underwent lumpectomy with sentinel lymph node biopsy (73%; *n* = 230). Sixty-seven percent performed adjuvant treatment (67%; *n* = 215) with chemotherapy and radiotherapy (38%) and hormone therapy (54%). The prevalence of participants presenting with symptoms of anxiety and depression (i.e., scoring above the mild threshold in PHQ-9 and GAD-7) was 63% (*n* = 211) and 65% (*n* = 217), respectively. Forty percent (*n* = 128) of the women had a psychiatric history. Considering HRQoL, the sample median global health status was 50 (IQR = 33; Table 1).

### 3.2. Digital Technology Use and eMH Self-Reported Knowledge

A majority of participants had access to the internet (78%; *n* = 263), mainly via domestic and/or mobile data (88%; *n* = 226), using it daily (85%; *n* = 217) on mobile devices (52%; *n* = 174) or personal computers (21%; *n* = 70). Most participant survivors searched for health-related information on the internet (65%; *n* = 219) without the support of a third party (55%; *n* = 184). The most-searched topics by these participants were: healthy lifestyle habits (43%; *n* = 145); BC risk factors and prevention (39%; *n* = 132); treatments adverse effects (39%; *n* = 131); BC signs and symptoms (34%; *n* = 115); BC research (33%; *n* = 111); survival and recurrence (27%; *n* = 91); and BC jargon (27%; *n* = 90). Twenty-six percent (*n* = 85) of participants used the internet to search for information about psychological problems. A minority used it to search for mental healthcare (8%; *n* = 26).

Regarding remote healthcare, 18% (*n* = 60) of the participants had used it. The most-used modalities by these participants were telephone consultations with physicians (13%; *n* = 42) and nurses (8%; *n* = 28), and SMS (4%; *n* = 12), e-mail (2%; *n* = 8) and chat (1%; *n* = 4) communications with physicians. Few participants had used such media to access psychological support. The modalities used were telephone (1%; *n* = 4) and e-mail (1%; *n* = 3). Medical/nursing videoconference consultations were rarely used, with 0.3% (*n* = 1) reporting using this. None of the participants had prior experience with videoconference counseling/psychotherapy or internet interventions. Self-help groups (1%; *n* = 2) and other forms of peer support (2%; *n* = 7) were seldom used.

Twenty-six percent (*n* = 88) of respondents were familiar with eMH. Nevertheless, 44% (*n* = 146) considered themselves skilled enough to use eMH. Fifty-four (*n* = 181) reported that they would be willing to use eMH if it was provided by their cancer center.

### 3.3. BCS’ Attitudes toward eMH

The ATIIS median score was 50.0 (IQR = 23), which indicates a neutral attitude toward eMH. An analysis of participants’ responses to ATIIS revealed that BCS tended to value eMH’s accessibility (50%; *n* = 169); the possibility of communicating with therapists online (49%; *n* = 164); and its convenience (46%; *n* = 155), anonymity (43%; *n* = 143), and empowerment potential (42%; *n* = 141). Around 52% (*n* = 174) of participants reported being willing to use eMH if diagnosed with a psychological condition. Conversely, uncertainty regarding the security (44%; *n* = 148), accuracy (45%; *n* = 152), and efficacy (52%; *n* = 173) of eMH was also reported. Finally, most participants perceived in-person treatment strategies as more efficacious (58%; *n* = 196), easier to acquire (60%; *n* = 201), and better for handling crises (63%; *n* = 210) than eMH.

### 3.4. Variables Associated with BCS’ Attitudes toward eMH

The relationship between ATIIS and age, distance from the reference center, time since diagnosis, and GAD-7, PHQ-9, QLQC30, and QLQBR23 scales scores were examined using Spearman rank correlations. Statistically significant weak negative correlations were found between ATIIS continuous scores and age (r_336_ = −0.206; *p* < 0.001); QLQC30 emotional (r_331_ = −0.136; *p* < 0.013), cognitive (r_331_ = −0.171; *p* < 0.002) and social functioning (r_331_ = −0.141; *p* < 0.010) scales; and the QLQBR23 body image scale (r_324_ = −0.190; *p* < 0.001). Significant weak positive correlations were found between ATIIS continuous scores and PHQ-9 (r_336_ = 0.148; *p* < 0.007) and GAD-7 (r_334_ = 0.114; *p* < 0.037).

χ^2^ tests and post hoc analyses were performed to explore associations between ATIIS quartiles and sociodemographic, clinical, and internet-related variables; eMH self-reported knowledge; and remote healthcare use. Differences between participants holding extreme attitudes toward eMH were also evaluated. To this end, percentiles were computed to identify participants who held highly negative (scores < Q1 = 37.5; *n* = 80; 24%) and highly positive (scores > Q3 = 60.9; *n* = 90; 27%) attitudes.

χ^2^ analyses revealed significant associations between education (χ^2^, *p* < 0.001); psychiatric history (χ^2^, *p* < 0.023); internet access (χ^2^, *p* < 0.001); internet use frequency (χ^2^, *p* < 0.001); social networks use (χ^2^, *p* < 0.001); online health information (χ^2^, *p* < 0.001) and online mental healthcare seeking (χ^2^, *p* < 0.001); remote healthcare use (χ^2^, *p* < 0.011); eMH self-reported knowledge (χ^2^, *p* < 0.001); and ATIIS quartiles (Table 2).

Considering education, participants who had a college degree were more likely to hold a positive attitude toward eMH. In contrast, respondents with 4–6 years of formal education were less likely to have a positive stance toward it. Similarly, participants with no psychiatric history were less likely to hold positive attitudes toward eMH. Respondents with a prior history of psychological or psychiatric support were more prone to evidencing positive attitudes toward eMH.

Concerning internet access, respondents with access to the internet were more likely to have a positive stance toward eMH. Conversely, subjects without access to the internet were less likely to endorse it. In addition, participants using the internet several times a day had a higher probability of holding a positive stance toward eMH than subjects not using the internet. The use of social networks also seemed to influence participants’ attitudes. Whereas respondents who used social networks were more likely to hold a positive stance toward eMH, participants who did not use them were more likely to present negative attitudes toward it.

Regarding online health information searching and online mental healthcare seeking, participants reporting such behaviors had a higher probability of presenting positive attitudes toward eMH. The opposite was also verified. Respondents who reported never to have searched for health information or sought mental healthcare online tended to hold a more negative attitude toward eMH. Participants reporting previous experience of remote healthcare use also tended to have a more positive stance toward eMH when compared with participants with no such experience.

Finally, awareness of eMH also appeared to influence BCS’ attitudes toward eMH. Whereas participants reporting to be moderately or completely familiar with the concept tended to present a more positive attitude toward eMH, respondents who were completely unaware of such an approach were less likely to endorse it.

No significant associations were found between ATIIS quartiles and demographic or clinical variables such as marital and professional status, area of residence, type of cancer treatment, disease staging, or ECOG. To further assess these associations while controlling for potential confounders, various multiple linear regression models (method: enter) were generated with age and education as fixed covariates. After adjustment, age; education; psychiatric history; GAD-7; social functioning; internet access; and internet use frequency were no longer significantly associated with ATIIS (cf., Table 3).

In the adjusted models (method: enter), a significant positive weak association between PHQ-9 and ATIIS continuous scores (β = 0.109, *p* < 0.041) was identified. These results suggest that more positive attitudes toward eMH were associated with higher depressive symptoms scores. Significant negative, weak associations were obtained between emotional (β = −0.129, *p* < 0.017), cognitive (β = −0.136, *p* < 0.013), and body image (β = −0.168, *p* < 0.002) functioning and ATIIS continuous scores, suggesting that better levels of functioning were associated with more negative attitudes toward eMH. Additionally, a significant positive weak impact of online health information searching (β = 0.240, *p* < 0.001) and mental healthcare (β = 0.180, *p* < 0.001) seeking was observed in attitudes. Prior experiences of such behaviors were weakly associated with better attitudes toward eMH. Likewise, social network use (β = 0.212, *p* < 0.001) and remote healthcare use (β = 0.107, *p* < 0.047) were positively weakly associated with ATIIS continuous scores. These data suggest that individuals who engage in social networks and participants with prior experience in remote healthcare are more prone to endorsing eMH than participants without such experience.

Finally, eMH self-reported knowledge (β = 0.332, *p* < 0.001) was weakly and positively associated with participants’ attitudes. Respondents who were familiar with eMH were more likely to hold positive attitudes.

## 4. Discussion

A neutral stance was observed in this study on BCS’ attitudes toward eMH. This finding is in line with previous studies in the general population [48,49] but contrasts with research targeting other clinical groups [50,51,52,53,54] or assessing BCS’ attitudes toward e-health in general [30,37,55,56,57]. Previous studies have yielded mixed results, reporting either a positive [30,50,52,54,55,56,57] or negative [37,51,58,59,60] attitude, and BCS have tended to endorse e-health. Only two other studies [29,30] have assessed BCS’ attitudes toward eMH specifically, having reported a positive stance. In perspective, our results suggest Portuguese BCS hold a cautious attitude toward eMH.

Uncertainty over the security, accuracy, and efficacy of eMH was reported by BCS participating in this study. Nevertheless, most reported a willingness to use eMH if provided by cancer centers, valuing its credibility, accessibility, and online contact with therapists. Aspects such as interventions’ ubiquity, anonymity, and empowerment were also viewed positively. Simultaneously, participants regarded in-person contacts as more efficacious than eMH. These findings echo previous research. In a prior study [37] focusing on cancer survivors, most participants acknowledged accessibility, flexibility, empowerment, and anonymity as advantages of internet-based treatments but still preferred in-person approaches. In another study [48], although most participants viewed internet interventions as useful and helpful, few considered them equivalent to face-to-face approaches. Concerns about the efficacy, safety, and privacy of eMH have also been documented previously [52,53]. Nevertheless, research on eMH views is scarce, and little is known about the variables associated with its acceptance and use.

Our results suggest attitudes toward eMH are statistically significantly associated with psychological distress. Participants presenting with more depressive symptoms were more likely to endorse eMH than those with mild symptoms or who were asymptomatic. No statistically significant associations were identified between attitudes and anxiety symptoms. Previous research [48,54,59] has reported divergent results. A plausible explanation for these findings might be related to depression being a better marker of higher negative affectivity and lower positive affectivity, better measuring psychological distress [61]. Nevertheless, the evidence base for these associations was weak, and their clinical significance might be limited, requiring further research.

Supporting this interpretation, lower emotional, cognitive, and body image functioning levels were associated with more positive attitudes toward eMH. BCS struggling with such changes tended to perceive an added value in eMH, possibly due to its non-stigmatizing nature. Results from a previous study [62] support this hypothesis. In that study, participants who preferred e-MH had significantly lower scores on emotional stability and higher stigma scores than those who preferred traditional services. In another study [63], internet treatment decreased primary-care patients’ perceived stigma toward seeking mental healthcare, suggesting it to be a more acceptable alternative to patients dreading its connotation.

Based on these previous findings, our results point to statistically significant associations between online health information and mental healthcare seeking and attitudes toward eMH. BCS adopting such behaviors were more likely to endorse eMH. Participants who reported using social networks also held more positive attitudes toward eMH. In line with our findings, Jansen et al. [57] documented positive attitudes toward self-management and eHealth in cancer survivors reporting unmet needs. Likewise, Wallin et al. [37] reported that cancer survivors who used internet searches to improve their health were more likely to prefer internet-based psychological interventions than individuals not doing this. These results attest to the importance of developing eMH programs targeting cancer survivors, particularly internet interventions, due to their self-care approach.

Another important finding was related to the statistically significant impact previous use of remote healthcare and eMH self-reported knowledge had on participants’ attitudes toward eMH. Respondents with prior experiences of remote healthcare and who could grasp the concept were more likely to endorse it than participants without such experience or awareness. Previous research has reported similar findings [64,65]. March et al. [65] identified prior experience using online services as a predictor of intentions to use self-help and therapist-assisted online services. In another study [48], associations between “e-awareness” and attitudes toward internet-based therapies did not reach statistical significance. Still, a high “e-awareness” was associated with a preference for guided internet interventions. Considering that eMH self-reported knowledge and experience may be moderators of attitudes toward eMH, promoting it might expedite eMH acceptance and use. Few studies tested this assertion. Nevertheless, research suggests that eMH acceptance can be substantially increased via texts [66,67] and video clips [68,69].

Finally, no significant associations were found between attitudes and sociodemographic or clinical variables. This is contrary to previous research identifying age [48], education, marital status [60], professional status [56], geographic location [65], type of treatment performed [57], and time since diagnosis [57], as predictors of eMH or e-Health acceptance. Nevertheless, many studies did not focus on BCS or their attitudes toward eMH. Alternatively, these results may reflect cultural differences or a sociodemographic shift regarding the use of digital technology for healthcare in this population.

### 4.1. Implications

Developing eMH programs targeting BCS appears to be a promising strategy to close the mental healthcare gap in oncology, given our sample’s high prevalence of psychological distress symptoms, neutral stance toward eMH, and readiness to use digital technologies for self-care. However, to increase this group’s acceptance and use of eMH, information campaigns focusing on the security and effectiveness of eMH seem to be required. Additional research is necessary to understand how BCS’ unmet care needs, mainly regarding psychological distress severity, may affect their adoption and usage of eMH.

### 4.2. Limitations

This study is not exempt from limitations. The cross-sectional study design, convenient recruitment strategy, and the fact that data collection took place before the COVID-19 pandemic in Portugal may hinder the generalizability of the results. Comparative research is required to understand cultural and geographical differences and the pandemic’s impact on BCS’ attitudes toward eMH. Moreover, although ATIIS’ attitudes section presents good psychometric properties, the two selected factors accounted for 61% of the variance explained. This is not uncommon in social sciences studies [70]. It indicates, however, that further research is necessary to identify attitudinal determinants. Future studies should build on established frameworks and adopt more nuanced methods, warranting comprehensive prediction models to be tested. Longitudinal studies assessing how attitudinal predictors affect eMH uptake and treatment outcomes would also provide valuable insight.

## 5. Conclusions

Developing eMH programs targeting BCS appears to be a promising strategy to close the mental healthcare gap in oncology. However, information campaigns focusing on the security and efficacy of eMH seem necessary to increase awareness of eMH among this group and drive acceptance and use of eMH. Additional research is also necessary to understand how BCS’ unmet care needs may affect their adoption and usage of eMH.

## Figures and Tables

**Table 1:** Participants’ characteristics (N = 336).

Variables		
Education, *n* (%)		
	No education	2 (1)
	4–6 school years	109 (32)
	9 school years	66 (20)
	12 school years	78 (23)
	University degree	81 (24)
Marital status, *n* (%)		
	Single	38 (11)
	Married/de facto relationship	225 (67)
	Divorced/Separated	43 (13)
	Widowed	30 (9)
Employment status, *n* (%)		
	Unemployed	52 (16)
	Active	107 (32)
	Sick leave	94 (28.)
	Retired	83 (25)
Surgery, *n* (%)		
	Not performed	68 (21)
	Lumpectomy	156 (49)
	Mastectomy	95 (29)
	Missing	17
Chemo/Radiotherapy, *n* (%)		
	Not performed	74 (23)
	Chemo and radiotherapy	120 (38)
	Only chemotherapy	87 (27)
	Only radiotherapy	38 (12)
	Missing	17
Hormone therapy, *n* (%)		
	Yes	172 (54)
	No	147 (46)
	Missing	17
Immunotherapy, *n* (%)		
	Yes	82 (25)
	No	237 (74)
	Missing	17
Disease staging, *n* (%)		
	0	12 (4)
	I	100 (32)
	II	79 (25)
	III	72 (23)
	IV	34 (11)
	Under determination	17 (5)
	Missing	22
ECOG, *n* (%)		
	0	198 (80)
	1	42 (17)
	2	8 (3)
	3	1 (0)
	Missing	87
PHQ-9, Median (IQR) ^1,2^		7.0 (8)
GAD-7, Median (IQR) ^1,3^		6.0 (7)
QLQ30, Median (IQR) ^1,4^		
Global Health Status		50 (33)
Functional scales		
Physical Functioning		73 (27)
Role Functioning		67 (50)
Emotional Functioning		67 (33)
Cognitive Functioning		83 (33)
Social Functioning		67 (33)
Symptoms scales		
Fatigue		33 (33)
Nausea and Vomiting		0.0 (17)
Pain		33 (50)
Single-item symptoms measures		
Dyspnea		0 (33)
Insomnia		33 (67)
Appetite loss		0.0 (33)
Constipation		0.0 (33)
Diarrhea		0.0 (33)
Financial Difficulties		33 (33)
QLQBR23, Median (IQR) ^1,5^		
Functional scales		83 (42)
Body Image		17 (33)
Sexual Functioning		
Symptoms scales		
Systemic Therapy Side Effects		19 (24)
Breast Symptoms		8 (25)
Arm Symptoms		22 (33)
Single-items measures		
Sexual Enjoyment		0 (33)
Future Perspective		33 (67)
Upset by Hair Loss		0 (0)

Abbreviations: IQR–Interquartile range, ECOG-Eastern Cooperative Oncology Group (ECOG) performance status; ^1^ Considering data are not normally distributed, measures’ scores are presented in medians; ^2^ Cronbach’s α = 0.88; ^3^ Cronbach’s α = 0.93; ^4^ Cronbach’s α = 0.89; ^5^ Cronbach’s α = 0.83.

**Table 2:** Variables associated with attitudes toward eMH (N = 336).

	ATIIS Score				Chi-Square Results	
	Quartile 1 (≤37.49)	Quartile 2 (37.50–49.99)	Quartile 3 (50.00–60.93)	Quartile 4 (≥60.94)	*p* Value	Chi-Square
Formal education					0.000	χ^2^ _12_ = 38.41
No degree	1 (0.9)	0 (−0.7)	1 (0.6)	0 (−0.9)		
1st/2nd cycles (4–6 school years)	33 (1.9)	26 (1.8)	39 (1.3)	11 (−4.8)		
3rd cycle (9 school years)	13 (−0.9)	11 (−0.4)	26 (1.7)	16 (−0.5)		
Secondary (12 school years)	20 (0.4)	13 (−0.5)	20 (−1.2)	25 (1.2)		
Tertiary (University degree)	13 (−1.9)	12 (−1.0)	18 (−2.0)	38 (4.7)		
Psychiatric history					0.023	χ^2^ _3_ = 9.56
No	41 (−1.1)	44 (2.1)	63 (1.3)	44 (−2.2)		
Yes	34 (1.1)	17 (−2.1)	33 (−1.3)	44 (2.2)		
Internet access					0.001	χ^2^ _3_ = 15.74
No	23 (1.7)	13 (−0.2)	30 (2.1)	7 (−3.8)		
Yes	57 (−1.7)	49 (0.2)	74 (−2.1)	83 (3.8)		
Internet use frequency					0.001	χ^2^ _12_ = 33.11
Never	25 (1.8)	20 (1.7)	29 (1.2)	6 (−4.5)		
Once a week or less	6 (1.3)	2 (−0.6)	5 (0)	3 (−0.7)		
2–3 times a week	5 (−0.2)	3 (−0.7)	9 (0.9)	6 (−0.1)		
Daily, at least once a day	24 (−0.6)	22 (0.5)	35 (0.2)	29 (−0.1)		
Daily, several times a day	20 (−1.5)	15 (−1.4)	26 (−1.8)	46 (4.6)		
Social networks use					0.000	χ^2^ _3_ = 34.77
No	39 (3.0)	27 (1.6)	42 (1.4)	9 (−5.8)		
Yes	41 (−3.0)	35 (−1.6)	62 (−1.4)	81 (5.8)		
Online health information seeking ^1^					0.000	χ^2^ _3_ = 29.93
No	41 (3.5)	27 (1.6)	37 (0.2)	12 (−5.0)		
Yes	39 (−3.5)	35 (−1.6)	67 (−0.2)	78 (5.0)		
Online mental healthcare seeking ^2^					0.000	χ^2^ _3_ = 21.43
No	77 (1.5)	60 (1.5)	100 (1.8)	73 (−4.6)		
Yes	3 (−1.5)	2 (−1.5)	4 (−1.8)	17 (4.6)		
Remote healthcare use ^3^					0.011	χ^2^ _3_ = 11.07
No	70 (1.4)	51 (0.0)	91 (1.7)	64 (−3.2)		
Yes	10 (−1.4)	11 (0.0)	13 (−1.7)	26 (3.2)		
eMH Self-reported knowledge ^4^					0.000	χ^2^ _12_ = 80.53
Completely disagree	36 (3.9)	18 (0.2)	20 (−2.4)	20 (−1.4)		
Moderately disagree	6 (−0.3)	5 (−0.1)	9 (0.1)	8 (0.2)		
Neither agree nor disagree	32 (0.5)	29 (1.7)	50 (2.7)	15 (−4.8)		
Moderately agree	5 (−2.9)	8 (−0.9)	21 (1.2)	22 (2.3)		
Completely agree	1 (−2.9)	2 (−1.9)	4 (−2.4)	25 (6.9)		

Abbreviations: eMH–eMental Health interventions; Notes: Adjusted standardized residual frequencies appear in parentheses after observed group frequencies; ^1^ Original wording: “Have you ever used the internet to search for information related to your health?”. Rated on a dichotomous scale: 0 = No, 1 = Yes; ^2^ Original wording: “Have you ever used the internet to seek psychological care or psychology professionals?”. Rated on a dichotomous scale: 0 = No, 1 = Yes; ^3^ Original wording: “Have you ever used the internet or the telephone to receive medical, nursing, or psychological care?”. Rated on a dichotomous scale: 0 = No, 1 = Yes; ^4^ Original wording: “I am familiar with the concept of psychological interventions carried out over the internet?”. Rated on a five-point scale: 1 = Completely disagree, to 5 = Completely agree.

**Table 3:** Results of the regression analysis (method: enter) on variables associated with attitudes toward eMH (N = 336).

	Model 1			Model 2			Model 3			Model 4			Model 5			Model 6			Model 7			Model 8			Model 9		
Variable	*B*	SE *B*	β	*B*	SE *B*	β	*B*	SE *B*	β	*B*	SE *B*	β	*B*	SE *B*	β	*B*	SE *B*	β	*B*	SE *B*	β	*B*	SE *B*	β	*B*	SE *B*	β
PHQ-9	0.38	0.19	0.11 *																								
Emotional functioning				−0.10	0.04	−0.13 *																					
Cognitive functioning							−0.10	0.04	−0.14 *																		
Body image										−0.12	0.04	−0.17 **															
Social networks													9.12	2.75	0.21 **												
Online health information seeking ^1^																10.33	2.85	0.24 **									
Online mental healthcare seeking ^2^																			13.79	4.14	0.18 **						
Previous use of remote healthcare ^3^																						5.71	2.86	0.11 *			
0 vs. eMH self-reported knowledge (Moderately Disagree) ^4^																									5.61	3.99	0.08
0 vs. eMH self-reported knowledge (Neither Agree nor Disagree) ^4^																									1.34	2.50	0.03
0 vs. eMH self-reported knowledge (Moderately Agree) ^4^																									13.43	3.14	0.24 **
0 vs. eMH self-reported knowledge (Totally Agree) ^4^																									28.70	3.87	0.41 **
*R* ^2^	0.08			0.09			0.09			0.10			0.10			0.11			0.10			0.08			0.24		
*F* for change in *R*^2^	5.02 (6329) **			5.39 (6324) **			5.49 (6324) **			6.11 (6317) **			6.24 (6329) **			6.63 (6329) **			6.26 (6329) **			4.98 (6329) **			11.48 (9326) **		

Abbreviations: eMH–EMental Health interventions; * *p* < 0.05. ** *p* < 0.01; ^1^ Original wording: “Have you ever used the internet to search for information related to your health?”. Rated on a dichotomous scale: 0 = No, 1 = Yes; ^2^ Original wording: “Have you ever used the internet to seek psychological care or psychology professionals?”. Rated on a dichotomous scale: 0 = No, 1 = Yes; ^3^ Original wording: “Have you ever used the internet or the telephone to receive medical, nursing or psychological care?”, Rated on a dichotomous scale: 0 = No, 1 = Yes; ^4^ Original wording: “I am familiar with the concept of psychological interventions carried out over the internet?”. Dummy coded.

## Data Availability

The data that support the findings of this study are available upon request.

## Supplementary Materials

The following supporting information can be downloaded at: https://www.mdpi.com/article/10.3390/healthcare11131920/s1, Table S1: ATIIS Version A (Portuguese); File S1: ATIIS development and psychometric properties assessment [46,50,57,71,72,73,74,75].
