# Supplementary material for: Breast Cancer Survivors’ Attitudes toward eMental Health: A Cross-Sectional Study
Source: Healthcare (Basel). 2023 Jul 3;11(13):1920. doi: 10.3390/healthcare11131920 (PMC10341406; doi:10.3390/healthcare11131920)
Supplement: Supplementary file 1 [file healthcare-11-01920-s001.zip › healthcare-2421271-supplementary.pdf]

Table S1: ATIIS Version A (Portuguese)

QUESTIONÁRIO DE UTILIZAÇÃO E ATITUDES FACE A PRESTAÇÃO DE CUIDADOS PSICOLÓGICOS SUPORTADOS PELA INTERNET (ATIIS) – VERSÃO A

Código do participante: _________________ Código da instituição: ____________

Data da visita/contacto: ___/___/______ (dia/mês/ano)

INSTRUÇÕES: Gostaríamos de conhecer alguns pormenores sobre a utilização que faz da internet e a sua opinião acerca da prestação de cuidados psicológicos através da internet.

A prestação de cuidados psicológicos através da internet consiste, geralmente, na implementação de um programa de tratamento psicológico específico (ex. terapia para tratamento da ansiedade), em que conteúdos educacionais acerca do alvo da intervenção (ex. ansiedade) bem como, estratégias para o tratamento ou gestão do mesmo (ex. técnicas de relaxamento) são disponibilizados de forma organizada num website (página na internet) ou aplicação móvel (app), podendo a implementação deste programa ser ou não acompanhada/supervisionada por um especialista em saúde mental.

Este questionário é breve e não levará mais de 10 minutos a completar. Responda por favor, às perguntas que se seguem, selecionando/clicando na opção de resposta que melhor se aplica ao seu caso. A informação fornecida é estritamente confidencial. Obrigada pela sua colaboração.

1. Tem, habitualmente, acesso à internet?

a. Não …………………………………………………………………………………………… ☐
b. Sim …………………………………………………………………………………………… ☐

2. Quando acede à internet que tipo de ligação utiliza mais frequentemente?

a. Não uso a internet ……………………………………………………………………….. ☐
b. Ligação pessoal (doméstica ou pacote de dados no telemóvel/tablet) …………………………… ☐
c. Ligação privada (local de trabalho e/ou outras instituições privadas) …………………………….. ☐
d. Ligações públicas (disponíveis em espaços públicos) ……………………………………………… ☐

3. Com que frequência usa a internet?

a. Não uso a internet ……………………………………………………………………….. ☐
b. Uma vez por semana ou menos ………………………………………………………….. ☐
c. 2 a 3 vezes por semana …………………………………………………………………… ☐
d. Diariamente, pelo menos uma vez por dia ………………………………………………… ☐
e. Diariamente, várias vezes por dia ……………………………………………………….. ☐

4. Que equipamento utiliza, mais frequentemente, para aceder à internet?

a. Não uso a internet ……………………………………………………………………….. ☐
b. Computador pessoal ………………………………………………………………………… ☐
c. Computador de terceiros (familiares, amigos, disponível no local de trabalho ou espaços públicos) ………………………………………………………………………………... ☐
d. Dispositivos móveis (Telemóvel/Tablet) ……………………………………………………… ☐

5. Tem um endereço de correio eletrónico (e-mail)?

a. Não .... ☐
b. Sim .... ☐

6. Costuma utilizar as redes sociais (Ex. Facebook, Skype, WhatsApp, etc.)?

a. Não .... ☐
b. Sim .... ☐

7. Alguma vez utilizou a internet para pesquisar informação relacionada com a sua saúde?

a. Não .... ☐
b. Sim .... ☐

8. Se sim, costuma fazê-lo sozinho(a) ou pede ajuda a terceiros?

a. Sozinho(a) .... ☐
b. Peço ajuda a terceiros (ex. amigos, familiares, colegas de trabalho, etc.) .... ☐

9. Se sim, quais os temas que costuma pesquisar (assinale todos os campos aplicáveis)?

a. Informação relacionada com hábitos de vida saudáveis... ☐
b. Informação relacionada com o cancro da mama:
   i. Prevenção e fatores de risco associados ao cancro da mama .... ☐
   ii. Sinas e sintomas da doença .... ☐
   iii. Exames diagnósticos e de estadiamento da doença .... ☐
   iv. Tratamentos e seus efeitos secundários (imediatos/tardios) .... ☐
   v. Consultas e seguimento da doença .... ☐
   vi. Sobrevivência e reaparecimento da doença .... ☐
   vii. Problemas psicológicos associados ao cancro da mama (ex. ansiedade, depressão, medo da recidiva, problemas de sono, problemas familiares, dificuldades sexuais, etc.) .... ☐
   viii. O que acontece após o fim dos tratamentos .... ☐
   ix. Programas de apoio a doentes oncológicos .... ☐
   x. Investigação sobre a doença .... ☐
   xi. Informação relativa a instituições de apoio a doentes oncológicos .... ☐
   xii. Definição de palavras que não entende .... ☐
c. Informação relacionada com outros problemas de saúde .... ☐
d. Informação relacionada com apoios da segurança social/seguros de saúde, etc .... ☐
e. Outros .... ☐

10. Por favor indique os recursos online (websites, apps, etc) por si mais frequentemente utilizados: ______________

11. Alguma vez utilizou a internet para entrar em contacto/receber apoio de outras pessoas com antecedentes de cancro da mama (ex. grupos de discussão na internet, redes sociais, etc.)?

a. Não ………………………………………………………….………….……………………… ☐
b. Sim ……………………………………………..……………………...………………………… ☐

12. Alguma vez utilizou a internet para procurar informação sobre problemas de saúde psicológica?

a. Não ………………………………………………………….………….……………………… ☐
b. Sim ……………………………………………..……………………................................... ☐

13. Alguma vez utilizou a internet para procurar cuidados psicológicos ou profissionais de Psicologia?

a. Não ………………………………………………………….………….……………………… ☐
b. Sim ……………………………………………..……………………................................... ☐

14. Alguma vez utilizou a internet ou o telefone para receber cuidados médicos, de enfermagem e/ou psicológicos?

c. Não ………………………………………………………….………….……………………… ☐
d. Sim ……………………………………………..……………………................................... ☐

15. Se sim, qual o tipo de cuidados recebidos (assinale todos os apoios que utilizou ou recebeu)?

a. Contacto com o meu médico assistente: ☐
   i. Via chamada telefónica
   ii. Via SMS
   iii. Via e-mail
   iv. Via Chat
b. Contacto com o meu psicólogo(a): ☐
   i. Via chamada telefónica
   ii. Via SMS
   iii. Via e-mail
   iv. Via Chat
c. Contacto com equipa de enfermagem: ☐
   i. Via chamada telefónica
   ii. Via SMS
   iii. Via e-mail
   iv. Via Chat
d. Consulta médica através da internet (efetuada à distância com apoio de programa para videoconferência) …………………………………………………………. ☐
e. Consulta psicológica através da internet (efetuada à distância com apoio de programa para videoconferência) …………………………………………………………. ☐
f. Consulta de enfermagem através da internet (efetuada à distância com apoio de programa para videoconferência) …………………………………………………………. ☐
g. Programa de apoio psicológico através da internet <u>sem apoio de um especialista</u> (website disponibilizando informação acerca das suas dificuldades físicas e psicológicas e informação acerca de como gerir essas dificuldades, sem acompanhamento de um profissional de saúde especializado) ………………………………………………………………………….................. ☐
h. Programa de apoio psicológico através da internet <u>com apoio de um especialista</u> (website disponibilizando informação acerca das suas dificuldades físicas e/ou psicológicas e ☐

informação acerca de como gerir essas dificuldades, com acompanhamento de um profissional de saúde especializado) ……………………………………………………………………………..

i. Grupo de autoajuda na internet (grupos de interajuda organizados e integrados por pessoas que partilham experiências semelhantes, com vista a encontrar soluções pela partilha e troca de informações) ………………………………………………………………………………………. ☐

j. Outros (consulta de blogs, chats, fóruns de discussão, redes sociais, etc.) ……………………….. ☐

As seguintes afirmações relacionam-se com o seu conhecimento acerca das intervenções psicológicas suportadas pela internet. Por favor, avalie cada uma das afirmações e responda de acordo com o seu grau de concordância.

	Discordo totalmente	Discordo moderadamente	Não concordo nem discordo	Concordo moderadamente	Concordo totalmente
16. Estou familiarizado(a) com o conceito de intervenções psicológicas realizadas através da internet.					
17. Tenho conhecimento de como funcionam as intervenções psicológicas realizadas através da internet.					

As seguintes afirmações relacionam-se com a utilização da internet e intervenções psicológicas suportadas pela internet, com o objetivo de melhorar o seu estado físico e psicológico.

Por favor, avalie cada uma das afirmações com base na sua experiência ou, caso não esteja familiarizado(a) com tais intervenções, partilhe as suas expectativas.

	Discordo totalmente	Discordo moderadamente	Não concordo nem discordo	Concordo moderadamente	Concordo totalmente
18. Considero ter capacidade para utilizar uma intervenção psicológica realizada através da internet.					
19. Se me visse confrontado com um problema de saúde psicológica estaria interessado(a) em utilizar uma intervenção psicológica disponibilizada através da internet.					
20. Considero que as intervenções psicológicas realizadas através da internet são eficazes.					

21. A internet é um meio seguro para receber apoio psicológico.					
22. A internet é um meio adequado para avaliar/acompanhar o meu estado psicológico e melhorias ao longo do tratamento.					
23. Ter acesso a um programa de apoio psicológico realizado através da internet far-me-ia sentir ter mais controlo sobre a minha saúde.					
24. Gostaria de manter contacto com especialistas em saúde mental através da internet.					
25. O facto de poder receber apoio psicológico através da internet de forma discreta e anónima (sem ter de me identificar) faz com que este tipo de tratamento seja interessante para mim.					
26. O facto de poder receber apoio psicológico através da internet sem ter que me deslocar a uma unidade de saúde faz com que este tipo de tratamento seja interessante para mim.					
27. Não me sinto seguro(a) em partilhar informação pessoal através da internet, mesmo que através de um website que cumpra os requisitos de segurança máximos (ex. os que são utilizados nos websites dos bancos).					
28. É para mim mais fácil dizer o que sinto através da internet do que pessoalmente.					
29. Sentir-me-ia mais confortável em receber apoio psicológico através da internet do que pessoalmente.					
30. Considero que receber apoio psicológico presencialmente por parte de um especialista é mais eficaz do que receber apoio psicológico através da internet.					
31. Considero que a aprendizagem de estratégias para lidar com os meus problemas é mais fácil com o apoio de um					

especialista, presencialmente, do que através da internet.					
32. O facto de as intervenções psicológicas realizadas através da internet me permitirem aceder a conteúdos informativos, registo de indicações dadas pelo psicólogo online, instruções para aplicar as técnicas de tratamento aprendidas, etc., a qualquer hora e em qualquer lugar, faz com que este tipo de tratamento seja interessante para mim.					
33. Numa situação de crise, recorreria mais facilmente a um especialista que me pudesse ajudar pessoalmente do que através da internet.					
34. Se a unidade de saúde onde sou seguido(a) disponibilizasse intervenções psicológicas através da internet para a minha condição, consideraria utilizá-las.					

File S1: ATIIS development and psychometric properties assessment

ATIIS is a self-developed questionnaire characterizing patients' and therapists' use and attitudes toward eMental Health (eMH). The questionnaire has two different versions tailored to psychologists and clients. While the psychologists' version has already been validated for the Portuguese population (α=.91) [46], the clients' version was adapted and validated for Breast Cancer Survivors (BCS) in the context of this research.

After performing a comprehensive literature review [50,57,71], a preliminary version of the questionnaire was submitted to the appraisal of researchers and clinicians within the fields of Oncology, and eMH and pilot tested with two BCS. ATIIS final version included 34 items assessing: 1) information relating to the use of digital technology for healthcare purposes (e.g., digital technology use and proficiency; contexts and purposes of use; 2) eMH self-reported knowledge of eMH interventions); and 2) attitudes toward eMH, i.e., cognitive, affective and behavioral predispositions of favor or disfavor [72] toward eMH interventions (e.g., efficacy and accessibility; security and confidentiality; and patient's empowerment and willingness to use it). As participants were not expected to be familiar with the concept of eMH, an introductory definition adapted from Barak et al. [73] was provided. The questions were asked in the form of dichotomous and multiple-choice questions and in the form of five-point Likert scales (1="Completely disagree"; 5="Completely agree"). A linear transformation was implemented to obtain standardized scores ranging from 0 to 100 with higher scores representing a more positive attitude toward eMH.

To test the psychometric properties of the attitudes section of ATIIS in the BCS population we explored its construct validity and reliability. An Exploratory Factor Analysis (EFA) based on the Principal Component Analysis (PCA) method and using a varimax rotation was conducted to study ATIIS underlying latent factors and determine its construct validity. The Kaiser-Meyer-Olkin (KMO) test and Bartlett's test of sphericity were calculated to measure sampling adequacy (confirmed if KMO value greater than .5) and appropriateness of the extracted factors (significant at $p<.05$), respectively. The whole study sample (N=336) was used in this analysis. A KMO=.92 confirmed the sampling adequacy and Bartlett's test of sphericity, χ^2 (136)=3955.49 ($P<.001$) indicated a possible statistically significant inter-relationship between variables and, therefore, confirmed the Factorial Analysis validity to perform factor reduction.

The initial EFA resulted in 3 factors with eigenvalues above Kaiser's criterion of 1. However, a scree plot analysis revealed inflexions compatible with the retention of two factors. Due to convergence with theory, two factors were retained for the final EFA. The initial model hypothesized that items would load on either a positive or a negative factor. Total variance explained by these two factors was 60.76% (factor one: 48.07% and factor two: 12.69%; unrotated solution) or (factor one: 44.85% and factor two: 15.91%; rotated solution) and items clustering on these two factors suggested that the questionnaire measures two dimensions, labelled as Positive Attitudes (PA; range of factor loadings: .467-.867) and Negative Attitudes (NA; range of factor loadings:

.300-.874). Items with factor loadings above r=.4 were considered acceptable [74]. The attitudes section final version included 16 items clustering in two dimensions labelled as "positive attitudes" and "negative attitudes". Scores on the negative items were reversed, and dimensions' scores were weighted, summed, and rescaled on a 100-point scale to simplify interpretation and obtain a continuous indicator of attitude towards eMH interventions. Higher scores indicated a more positive attitude. The loading factors for the ATIIS attitudes section are presented below (see File S1-Table 1).

Finally, ATIIS' reliability was assessed via the computation of Cronbach's α. ATIIS total scale revealed excellent (α=.93) internal consistency and its subscales, positive (α=.94) and negative attitudes (α=.82), showed good internal consistency [75].

File S1-Table 1: ATIIS attitudes section factor analysis (rotated component matrix)

Scale: 1=Completely disagree, to 5=Completely agree.

	**Factor Loading**	
**Item**	**1**	**2**
**Factor 1: Negative attitudes (α=.818)**		
14. I believe it is easier to learn strategies to deal with my problems with the support of a specialist, in person, than through the internet.	-0.063	0.874
13. I believe that receiving psychological support provided in person by an expert is more efficacious than receiving psychological support via the internet.	-0.090	0.871
18. In a crisis, I would more easily turn to a specialist who could help me in person than through the internet.	-0.084	0.771
**Factor 2: Positive attitudes (α=.944)**		
6. Having access to a psychological support program carried out over the internet would make me feel more in control of my health.	0.867	-0.170
7. I would like to keep in touch with mental health experts over the internet.	0.836	-0.121
9. The fact that I can access psychological support via the internet, without having to commute to a healthcare facility makes this type of treatment interesting to me.	0.826	-0.122
2. If I were faced with a psychological health problem, I would be interested in using a psychological intervention made available through the internet.	0.815	-0.128
17. If the healthcare service where I am being followed provided psychological interventions over the internet, I would consider using it.	0.807	-0.086
5. The internet is an adequate mean to assess/monitor my psychological state and improvements throughout the treatment.	0.805	-0.217

8. The fact that I can access psychological support via the internet discreetly and anonymously (without having to identify myself) makes this type of treatment interesting to me.	0.802	-0.117
4. The internet is a safe means to receive psychological support.	0.794	-0.177
16. The fact that psychological interventions delivered via the internet allow me to access informative content, indications provided by my therapist, instructions on how to implement treatment strategies, etc., anytime, anywhere, makes this type of treatment interesting to me.	0.793	-0.081
3. I believe psychological interventions delivered via the internet are efficacious.	0.782	-0.111
1. I believe I have the necessary skills to use an internet-delivered psychological intervention.	0.699	-0.058
12. I would feel more comfortable receiving psychological support via the internet, than in person.	0.528	-0.454
11. It is easier for me to express how I feel over the internet than in person.	0.467	-0.308

References:

46 Mendes-Santos, C.; Weiderpass, E.; Santana, R.; Andersson, G. Portuguese Psychologists' Attitudes Toward Internet Interventions: Exploratory Cross-Sectional Study. *JMIR Ment. Health* **2020**, *7*, e16817. https://doi.org/10.2196/16817.

71 J. Schröder, L. Sautier, L. Kriston, T. Berger, B. Meyer, C. Späth, U. Köther, Y. Nestoriuc, J.P. Klein, S. Moritz, Development of a questionnaire measuring Attitudes towards Psychological Online Interventions-the APOI, J Affect Disord. 187 (2015) 136–141. https://doi.org/10.1016/j.jad.2015.08.044.

50 Yao, X.-Y.; Li, Z.; Arthur, D.; Hu, L.-L.; Cheng, G. The feasibility of an internet-based intervention for Chinese people with mental illness: A survey of willingness and attitude. *Int. J. Nurs. Sci.* **2014**, *1*, 28–33. https://doi.org/10.1016/j.ijnss.2014.02.008.

57 F. Jansen, C.F. van Uden-Kraan, V. van Zwieten, B.I. Witte, I.M. Verdonck-de Leeuw, Cancer survivors' perceived need for supportive care and their attitude towards self-management and eHealth, Support Care Cancer. 23 (2015) 1679–1688. https://doi.org/10.1007/s00520-014-2514-7.

72 I. Ajzen, Nature and operation of attitudes, Annu Rev Psychol. 52 (2001) 27–58. https://doi.org/10.1146/annurev.psych.52.1.27.

73 A. Barak, B. Klein, J.G. Proudfoot, Defining internet-supported therapeutic interventions, Ann Behav Med. 38 (2009) 4–17. https://doi.org/10.1007/s12160-009-9130-7.

74 K. Pituch, J. Stevens, Applied Multivariate Statistics for the Social Sciences: Analyses with SAS and IBM's SPSS, Sixth Edition, Routledge, 2016. https://www.routledge.com/Applied-Multivariate-Statistics-for-the-Social-Sciences-Analyses-with-SAS/Pituch-Stevens/p/book/9780415836661 (accessed on 4 March, 2021).

75 SPSS for Windows Step by Step: A Simple Guide and Reference, 11.0 Update - Darren George, Paul Mallery - Google Livros, (n.d.). https://books.google.pt/books/about/SPSS_for_Windows_Step_by_Step.html?id=AghHAAAAMAAJ&redir_esc=y (accessed on 8 March, 2021).
